# Thoracic empyema with scarlatiniform rash and acral desquamation: a case report

**DOI:** 10.1186/1757-1626-2-9384

**Published:** 2009-12-22

**Authors:** John Scott Baird, Ivona Sediva

**Affiliations:** 1 Department of Pediatrics, St Vincents Hospital and Medical Center, 3959 Broadway New York, NY, USA

## Abstract

A 5 year old girl with thoracic empyema developed a scarlatiniform rash and acral desquamation. Cultures from blood, throat, and pleural fluid all grew Streptococcus pyogenes, a common etiologic agent of pediatric thoracic empyema. The presence of a scarlatiniform rash and acral desquamation in children with a thoracic empyema may help identify the causative organism.

## Background

The incidence of thoracic empyema complicating bacterial pneumonia in children appears to be increasing[1,2]; it is commonly associated with Streptococcus pneumoniae, Staphylococcus aureus, and Streptococcus pyogenes[1,3-5]. Common signs and symptoms are non-specific and include those related to the underlying pneumonia, with persistent fever and dyspnea. We report a 5 year old girl with thoracic empyema who developed a scarlatiniform rash and acral desquamation, and review the relevant literature.

## Case report

**Figure 1:**
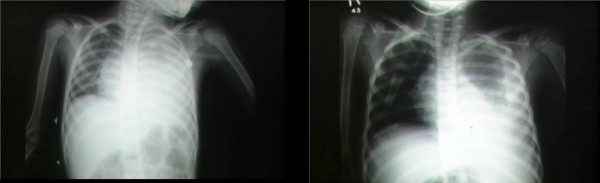
**Chest roentgenogram of child with thoracic empyema on hospital days 1 (1A) and 6 (1B)**.

The Integrated Scientific and Ethical Review Board of St Vincent Catholic Medical Center exempted this case report from review; parental consent was obtained for the photographs. A 5 year old girl was admitted with 5 days of fever to 40.0 degrees C, non-productive cough and vomiting. There was poor oral intake for 3 days, with tachypnea and left-sided chest pain for 2 days prior to admission. On presentation she was in moderate respiratory distress with intercostal retractions and nasal flaring. Her temperature was 39.5 degrees C, heart rate 173/minute, respiratory rate 56/minute, blood pressure 95/64, and transcutaneous oxygen saturation 91% on room air. Her pharynx was erythematous without exudate. There were decreased breath sounds, dullness to percussion over the lower 2/3 of the left lung, and bronchial breath sounds above the level of dullness. Laboratory results on admission included: white blood cells 2.9 × 10^9^/L (39% neutrophils, 23% bands and 14% lymphocytes), erythrocyte sedimentation rate (ESR) 75 mm/hr, sodium 132 mmol/L, blood urea nitrogen 45 mg/dL, creatinine 1.9 mg/dL, lactic acid 4.3 mmol/L, total protein 4.7 g/dL, albumin 2.3 g/dlL, calcium 6.4 mg/dL, lactate dehydrogenase 799 IU/L and anti-streptolysin O<100 IU/mL, with an arterial blood gas (while she was receiving oxygen): pH 7.32, PaCO_2 _16 mm Hg, PaO_2 _97 mmHg, and bicarbonate 8 mmol/L. A chest roentgenogram showed complete opacity of the left hemithorax with contralateral mediastinal shift (Figure 1A). Computed tomography of the chest confirmed the presence of a left pleural effusion with compressive atelectasis of the left lung. Pleural fluid analysis revealed an exudate with empyema (white blood cells 250,000/microL, glucose 24 mg/dL, total protein 2.5 g/dL, pH 7.22, lactate dehydrogenase> 21,500 IU/L, and gram stain with many gram positive cocci and few white blood cells). A thoracostomy tube was inserted in the left hemithorax. She was treated with crystalloid fluids, oxygen and broad spectrum antibiotics (vancomycin, imipenem, and clarithromycin).

**Figure 2:**
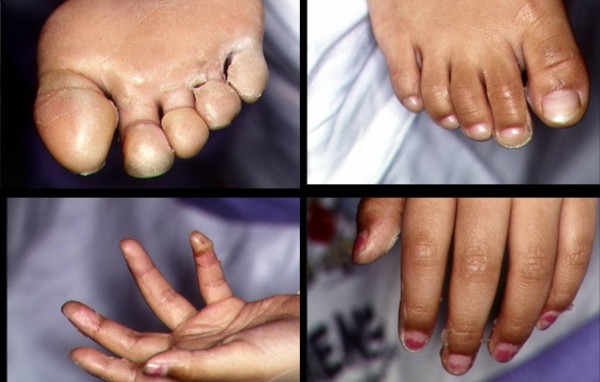
**Desquamation of the feet (2A, 2B) and hands (2C, 2D)**.

On the second hospital day she developed a mildly erythematous, sandpaper-like maculopapular rash over her trunk. Three days later desquamation of her toes was noted which progressed to involve her fingers and perineum (Figure 2). Cultures of blood, throat, and pleural fluid grew Streptococcus pyogenes, and antibiotics were changed to penicillin G. Total thoracostomy tube drainage was 565 cc over 6 days. She continued to have daily fever spikes to 40.6 degrees C. Repeat chest roentgenogram on hospital day 6 (Figure 1B) showed improved aeration in the left lung, and computed tomography scan on hospital day 9 showed near complete resolution of the left pleural fluid collection with interval appearance of a cavitary lesion in the left lung (2.5 × 1 cm) as well as several small loculated fluid collections. Antibiotic coverage was then broadened for several days, though follow-up blood cultures were sterile. Fever and an elevated ESR with leukocytosis persisted for 13 days after starting therapy.

The patient's condition gradually improved and she was discharged home after 3 weeks, and received another 3 weeks of antibiotics at home. Four months later a repeat chest roentgenogram was normal. The patient remained healthy and asymptomatic at follow-up examination 1 year later.

## Discussion

Efforts to isolate an organism are unsuccessful in at least 40 to 50% of all children with complicated parapneumonic effusions, including empyema[3,6,7]; Streptococcus pyogenes is likely responsible in some of these cases[3]. The association between thoracic empyema due to Streptococcus pyogenes and scarlatiniform rash with acral desquamation has been noted occasionally[8-10], and we suggest that this association may help identify Streptococcus pyogenes as the etiologic agent, particularly when culture results are unavailable. The rash of scarlet fever with subsequent acral desquamation is associated not just with pharyngitis, but may also follow systemic infections with Streptococcus pyogenes in children, including pneumonia[9,11], bacteremia[12,13], septic arthritis[13], Streptococcal toxic shock syndrome[14], and necrotizing fasciitis and cellulitis[11]. Streptococcus pyogenes toxins responsible for the rash and desquamation are pyrogenic exotoxins produced by certain strains of the bacteria. We found no reports of scarlatiniform rash or acral desquamation associated with Staphylococcus aureus or Streptococcus pneumoniae and pediatric thoracic empyema or pneumonia, though exfoliative toxins produced by certain strains of Staphylococcus aureus may also be responsible for a scarlatiniform rash and subsequent desquamation[15].

Resolution of pediatric thoracic empyema associated with Streptococcus pyogenes is often slow, as in our patient: fever, an elevated ESR, and leukocytosis may persist for several weeks in spite of adequate treatment[16,17], though the outcome is usually good. Initial management of pediatric empyema includes tube thoracostomy and empiric antibiotic therapy with antimicrobials effective against Staphylococcus aureus and Streptococcus pneumoniae; these regimens generally provide adequate coverage of Streptococcus pyogenes. Further therapy for pediatric thoracic empyema must be individualized, as there is little consensus on the precise role for surgical interventions, including video-assisted thoracoscopic surgery, or fibrinolysis[2].

## Abbreviations

ESR: erythrocyte sedimentation rate.

## Consent

Written informed consent was obtained from a parent for publication of this case report and accompanying images. A copy of the written consent is available for review.

## Competing interests

The authors declare that they have no competing interests.

## Authors' contributions

Both authors confirm that that each participated equally in the preparation of this manuscript, including the literature review, the clinical case presentation, and the discussion.
